# Supplementary material for: The solution structures of higher-order human telomere G-quadruplex multimers
Source: Nucleic Acids Res. 2021 Jan 19;49(3):1749–68. doi: 10.1093/nar/gkaa1285 (PMC7897503; doi:10.1093/nar/gkaa1285)
Supplement: gkaa1285_Supplemental_File [file gkaa1285_supplemental_file.pdf]

Supplementary Material: The solution structures of higher-order human telomere G-quadruplex multimers

Robert C. Monsen[1], Srinivas Chakravarthy[2], William L. Dean[3], Jonathan B. Chaires[1,3,4,*], and John O. Trent[1,3,4,*]

[1] Department of Biochemistry & Molecular Genetics, University of Louisville Medical School, Louisville, KY, 40202, USA

[2] The Biophysics Collaborative Access Team (BioCAT), Department of Biological Chemical and Physical Sciences, Illinois Institute of Technology, Chicago, IL, 60616, USA

[3] James Graham Brown Cancer Center, University of Louisville Medical School, Louisville, KY, 40202, USA

[4] Department of Medicine, University of Louisville Medical School, Louisville, KY, 40202, USA

[*] To whom the correspondence should be addressed: Tel: 1.502.852.2194; Fax: 1.502.852.7979; Email: john.trent@louisville.edu

[*] Correspondence may also be addressed to: Tel: 1.502.852.1172; Fax: 1.502.852.7979; Email: j.chaires@louisville.edu

Present Address: Clinical Translational Research Building, 505 S. Hancock St, University of Louisville Medical School, Louisville, KY, 40202, USA

TABLE AND FIGURE LEGENDS

Table S1. Tabulated collection parameters, data reduction methods, and data analyses for small-angle X-ray scattering data.

Figure S1. Guinier analyses of 2JSL, Tel48, Tel72, and Tel96 (left) with fit overlaid in yellow for each sequence and (right) residuals of fits. Guinier fit results are tabulated in Table S1.

Figure S2. Sedimentation velocity analysis of higher-order telomere sequences. (Top) Analysis of Tel72 and Tel96 concentration dependence on sedimentation. Extrapolation to the Y-axis gives the infinite dilution $S_{20,w}$ values. (Bottom) representative C(s) vs. $S_{20,w}$ distributions for 2JSL, Tel48, Tel72, and Tel96.

Figure S3. Additional results of Tel48 SAXS atomistic modeling efforts shown in **Figure 2** of main text. (A-B) scatter plots of calculated radii of gyration and sedimentation coefficients for hybrid-11 (A) and hybrid-22 (B) with MD-derived values shown in light gray and aMD-derived values in dark gray. The inset dashed red and blue lines represent the experimentally measured values for sedimentation coefficient and radius of gyration, respectively. The outer histograms represent the distributions of values from both MD and aMD snapshots combined. The histograms indicate that the major sampled conformations in both cases are much more compact than would be expected from either SAXS or AUC analyses.

Figure S4. Comparison of the Tel48 (cyan) and Tel50 (tan) hybrid-12 conformers. The tan hybrid-12 conformer was taken from an earlier report by Petraccone et al.(1) and the cyan hybrid-12 is the model derived here from EOM (bottom right-most conformer in **Figure 2D**). The pair-wise residue RSMD is 1.6 Å as determined by the matchmaker module of UCSF Chimera v1.12. Potassium is shown as purple spheres and is derived from the Petraccone model (EOM hybrid-12 potassium is hidden).

Figure S5. Results of Tel96 atomistic modeling efforts. Depicted are the four hybrid-2122 conformers derived from EOM analysis of the 100 ns MD simulations with their respective weights (as % of the reconstructed scattering curve). All conformers are arranged with their 5' ends at the top of the figure.

Figure S6. Normalized CD spectra of Tel48 compared to various flanking residue and internal mutant sequences.

Figure S7. Violin plots of residuals obtained as the difference between experimental and theoretical CD reconstruction curves for Tel48, Tel72, and Tel96. The residuals are plotted from best (left) to worst (right) based on residual sum of squares analysis in Origin 2020.

(a) Sample Details.

	2JSL	Tel48	Tel72	Tel96
Organism	synthetic	synthetic	synthetic	synthetic
Source	IDT	IDT	IDT	IDT
Extinction coefficient (nearest neighbor approximation) (M^{-1} cm^{-1})	253100	489000	733400	974870
vbar (cm^3/g) (estimate)	0.55	0.55	0.55	0.55
M from chemical composition (Da)	7879	15212	22849	30486
SEC-SAXS column, 10 x 300 Superdex 75				
Loading concentration (mg/mL)	7.0	13.0	10.0	6.0
Injection volume (µL)	300	300	250	440
Flow rate (mL/min)	0.7	0.7	0.7	0.7
Solvent (solvent blanks taken from SEC flow through prior to elution of protein)	8 mM PO_4^{2-}, 185 mM KCl, 1 mM EDTA, pH 7.2	8 mM PO_4^{2-}, 185 mM KCl, 1 mM EDTA, pH 7.2	8 mM PO_4^{2-}, 185 mM KCl, 1 mM EDTA, pH 7.2	8 mM PO_4^{2-}, 185 mM KCl, 1 mM EDTA, pH 7.2

(b) SAXS data-collection parameters.

Instrument/data processing	BioCAT facility at the Advanced Photon Source beamline 18ID with Pilatus3 1M (Dectris) detector
Wavelength (Å)	1.033
Beam size (µm)	150 (h) x 25 (v)
Camera length (m)	3.5
q measurement range ($Å^{-1}$)	0.004-0.4
Absolute scaling method	N/A
Normalization	To incident intensity, by ion chamber counter
Monitoring for radiation damage	Automated frame-by-frame comparison of relevant regions
Exposure time, number of exposures	0.5 s exposure time with a 2s total exposure period (0.5 s on, 1.5 s off) of entire SEC elution

Sample configuration	SEC-SAXS. Size separation by an AKTA Pure with a Superdex 75 Increase 10/300 GL column. SAXS data measured in a 1.5 mm ID quartz capillary
Sample temperature (°C)	20

(c) Software employed for SAXS data reduction, analysis, and interpretation.	
SAXS data reduction	Radial averaging; frame comparison, averaging, and subtraction done using BioXTAS RAW 1.6.3 (Hopkins et al. 2017(2))
Extinction coefficient estimate	Nearest neighbor approximation
Basic analyses: Guinier, P(r), V_p	Guinier fit, Kratky analysis, and molecular weight using BioXTAS RAW 1.6.3, P(r) function using PRIMUSqt (ATSAS v2.8.4(3))
Shape/bead modelling	DAMMIF (Franke & Svergun, 2009) via ATSAS online (https://www.embl-hamburg.de/biosaxs/atsas-online/)
Atomic structure modelling	CRYSOL from PRIMUSqt in ATSAS v2.8.4(Svergun et al., 1995(4))
Three-dimensional graphic model representations	UCSF Chimera v1.11

(d) Structural parameters.

Guinier analysis	2JSL	Tel48	Tel72	Tel96
I(0) (cm^{-1})	0.00957 ± 0.00002	0.0318 ± 0.00002	0.003742 ± 0.000005	0.0202 ± 0.0000406
R_g (Å)	12.41 ± 0.05	19.23 ± 0.03	25.37 ± 0.06	31.68 ± 0.13
qmin (Å^{-1})	0.014	0.009	0.007	0.007
qR_g max	1.27	1.33	1.32	1.17
Coefficient of correlation, R^2	0.972	0.998	0.996	0.996
M from volume of correlation, V_c (ratio to predicted)	6600 (0.84)	16100 (1.06)	23500 (1.03)	30900 (1.01)
P(r) analysis (GNOM)				
I(0) (cm^{-1})	0.00954 ± 0.00002	0.032 ± 0.00002	0.00376 ± 0.000005	0.0203 ± 0.00004
Rg (Å)	12.33 ± 0.03	19.69 ± 0.03	26.01 ± 0.06	32.65 ± 0.10
Dmax (Å)	38	65	87	109
χ^2	0.95	1.76	0.84	1.1

Porod volume (Å^{-3}) (ratio V_p/calculated M)	9040 (1.15)	16100 (1.06)	26300 (1.15)	32700 (1.07)

(e) Shape model-fitting results

	2JSL	Tel48	Tel72	Tel96
Ambimeter (default parameters)				
Number of compatible shape categories, ambiguity score	19, 1.279	634, 2.802	712, 2.852	644, 2.809
3D reconstruction	potentially unique	highly ambiguous	highly ambiguous	highly ambiguous
DAMMIF (default parameters, 20 calculations)				
q range for fitting (Å^{-1})	-	-	0.0088-0.3146	0.0054-0.2539
Symmetry, anisotropy assumptions	-	-	P1, prolate	P1, prolate
NSD (standard deviation), No. of clusters	-	-	1.204 (0.082), 4	1.126 (0.095), 11
χ^2	-	-	1.166	1.167
Resolution (from SASRES) (Å)			31 ± 3	38 ± 3
DAMMIN (default, slow)				
q range for fitting (Å^{-1})	0.014-0.3488	0.0115-0.3488	-	-
Symmetry, anisotropy assumptions	P1, none	P1, prolate	-	-
χ^2, CORMAP P-values	1.508, 0.1322	1.713, 0.249	-	-
Constant adjustment to intensities	8.46E-05	0.00E+00	-	-

(f) Atomistic modelling.

Crystal structures/atomic coordinate files	PDB ID: 2JSL	Modeled	Modeled	Modeled
q range for modelling	0.01-0.3	0.006-0.3	0.007-0.3	0.0054-0.2500
EOM GAJOE 2.1 (min ensembles = 1, max = 20, default parameters)				
χ^2	-	1.81	1.09	1.154

Rflex (random) / Rsigma	-	79.26 (88.83) / 0.62	79.96 (85.17) / 0.97	79.80 (86.06) / 1.39
Constant subtraction	-	0	0	0
No. of representative structures	-	6	4	4
Final ensemble Rg (Å), Dmax (Å)	-	19.58, 65.62	25.78, 82.65	32.11, 103.18
CRYSOL (single model, default parameters)				
χ^2	1.20	1.82	1.81	2.08
Predicted Rg (Å)	12.3	19.82	25.74	32.63
Dro (optimal hydration shell contrast), Ra (optimal atomic group radius (Å)	0.060, 1.760	0.065, 1.800	0.045, 1.400	0.075,1.400
(g) SASBDB IDs for data and models.				
ID	SASDKF3	SASDKG3	SASDKH3	SASDKJ3

Table S1. Tabulated collection parameters, data reduction methods, and data analyses for small-angle X-ray scattering data.

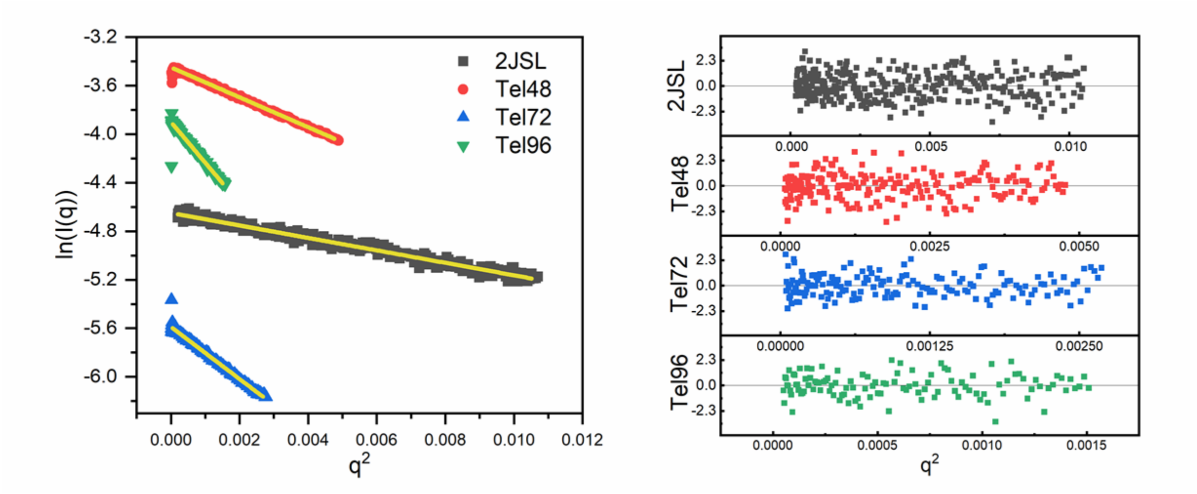

Figure S1. Guinier analyses of 2JSL, Tel48, Tel72, and Tel96 (left) with fit overlaid in yellow for each sequence and (right) residuals of fits. Guinier fit results are tabulated in Table S1.

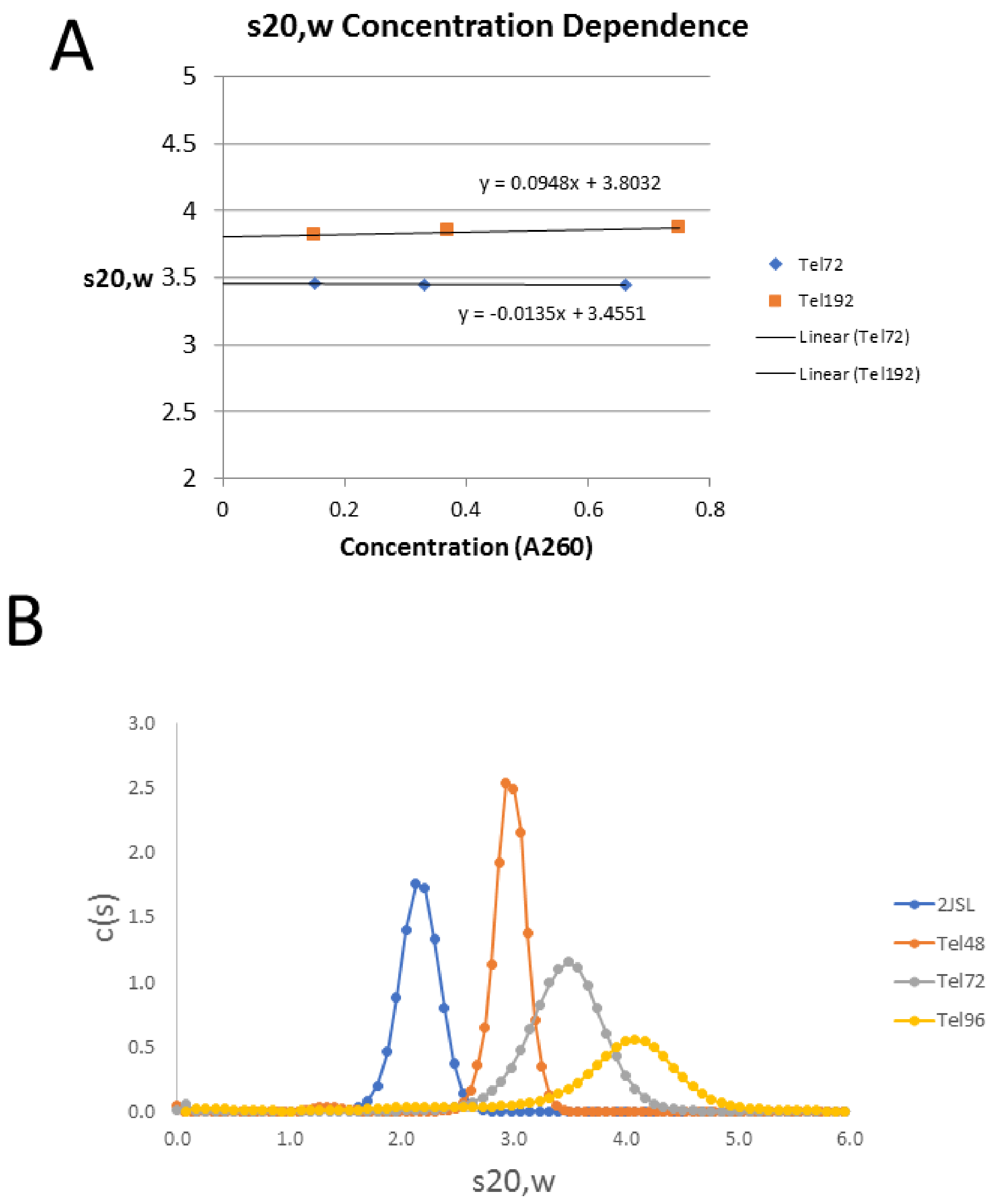

Figure S2. Sedimentation velocity analysis of higher-order telomere sequences. (Top) Analysis of Tel72 and Tel96 concentration dependence on sedimentation. Extrapolation to the Y-axis gives the infinite dilution $S_{20,w}$ values. (Bottom) representative C(s) vs. $S_{20,w}$ distributions for 2JSL, Tel48, Tel72, and Tel96.

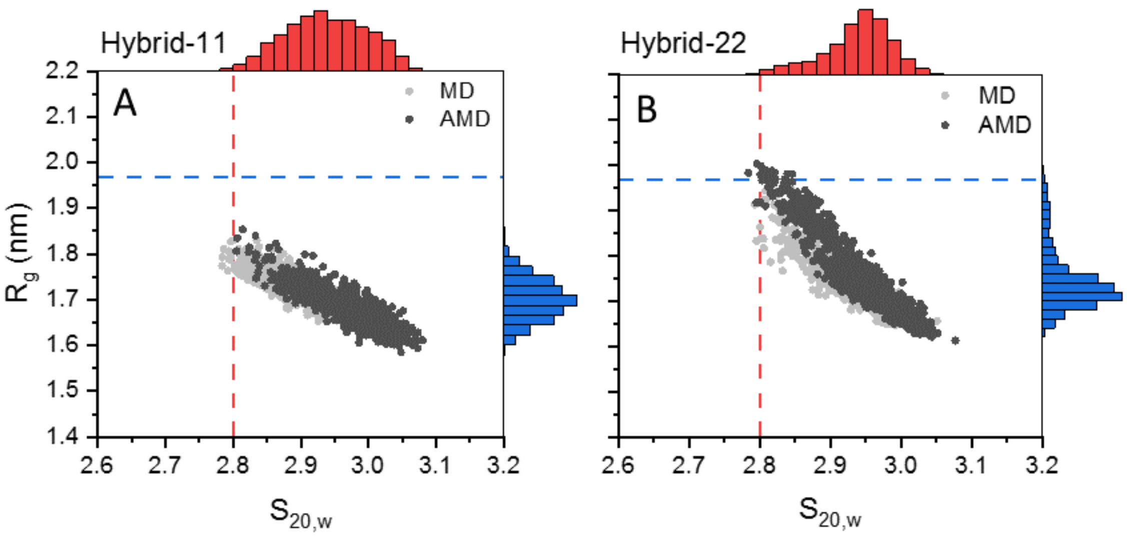

Figure S3. Additional results of Tel48 SAXS atomistic modeling efforts shown in **Figure 2** of main text. (A-B) scatter plots of calculated radii of gyration and sedimentation coefficients for hybrid-11 (A) and hybrid-22 (B) with MD-derived values shown in light gray and aMD-derived values in dark gray. The inset dashed red and blue lines represent the experimentally measured values for sedimentation coefficient and radius of gyration, respectively. The outer histograms represent the distributions of values from both MD and aMD snapshots combined. The histograms indicate that the major sampled conformations in both cases are much more compact than would be expected from either SAXS or AUC analyses.

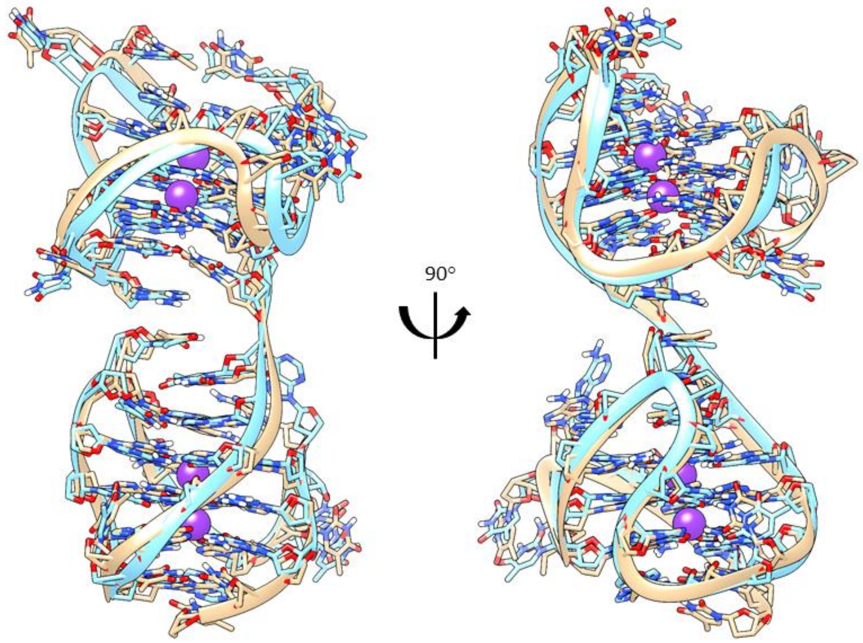

Figure S4. Comparison of the Tel48 (cyan) and Tel50 (tan) hybrid-12 conformers. The tan hybrid-12 conformer was taken from an earlier report by Petraccone et al.(1) and the cyan hybrid-12 is the model derived here from EOM (bottom right-most conformer in **Figure 2D**). The pair-wise residue RSMD is 1.6 Å as determined by the matchmaker module of UCSF Chimera v1.12. Potassium is shown as purple spheres and is derived from the Petraccone model (EOM hybrid-12 potassium is hidden).

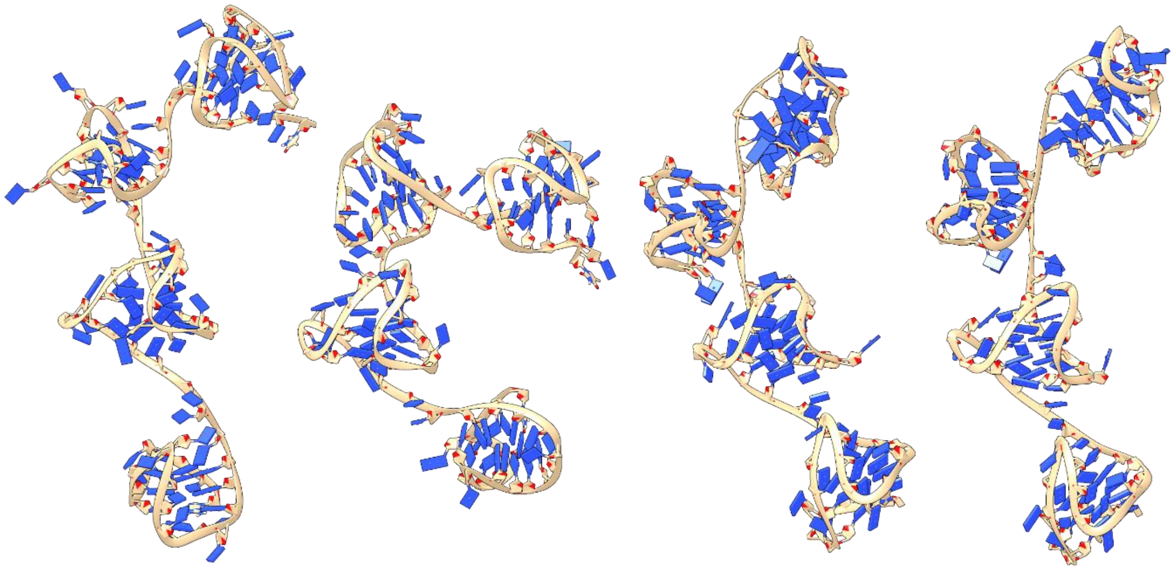

Figure S5. Results of Tel96 atomistic modeling efforts. Depicted are the four hybrid-2122 conformers derived from EOM analysis of the 100 ns MD simulations with their respective weights (as % of the reconstructed scattering curve). All conformers are arranged with their 5' ends at the top of the figure.

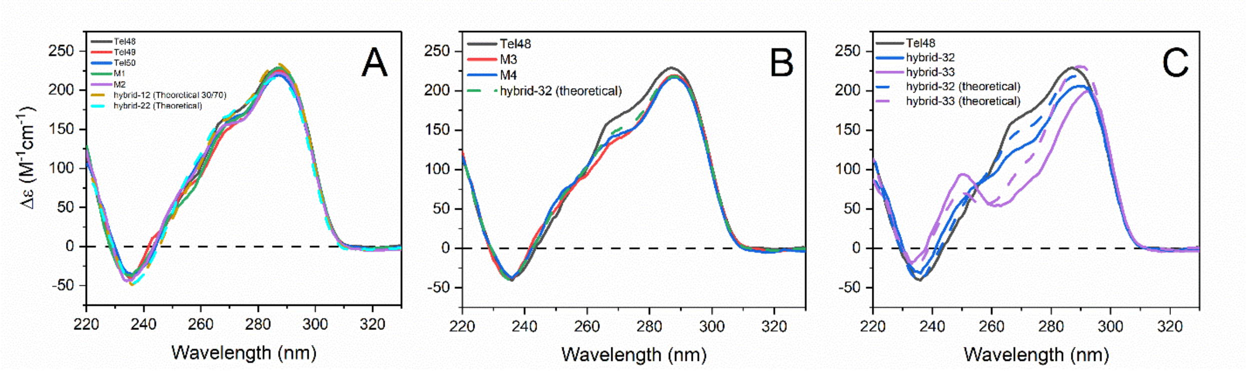

Figure S6. Normalized CD spectra of Tel48 compared to various flanking residue and internal mutant sequences.

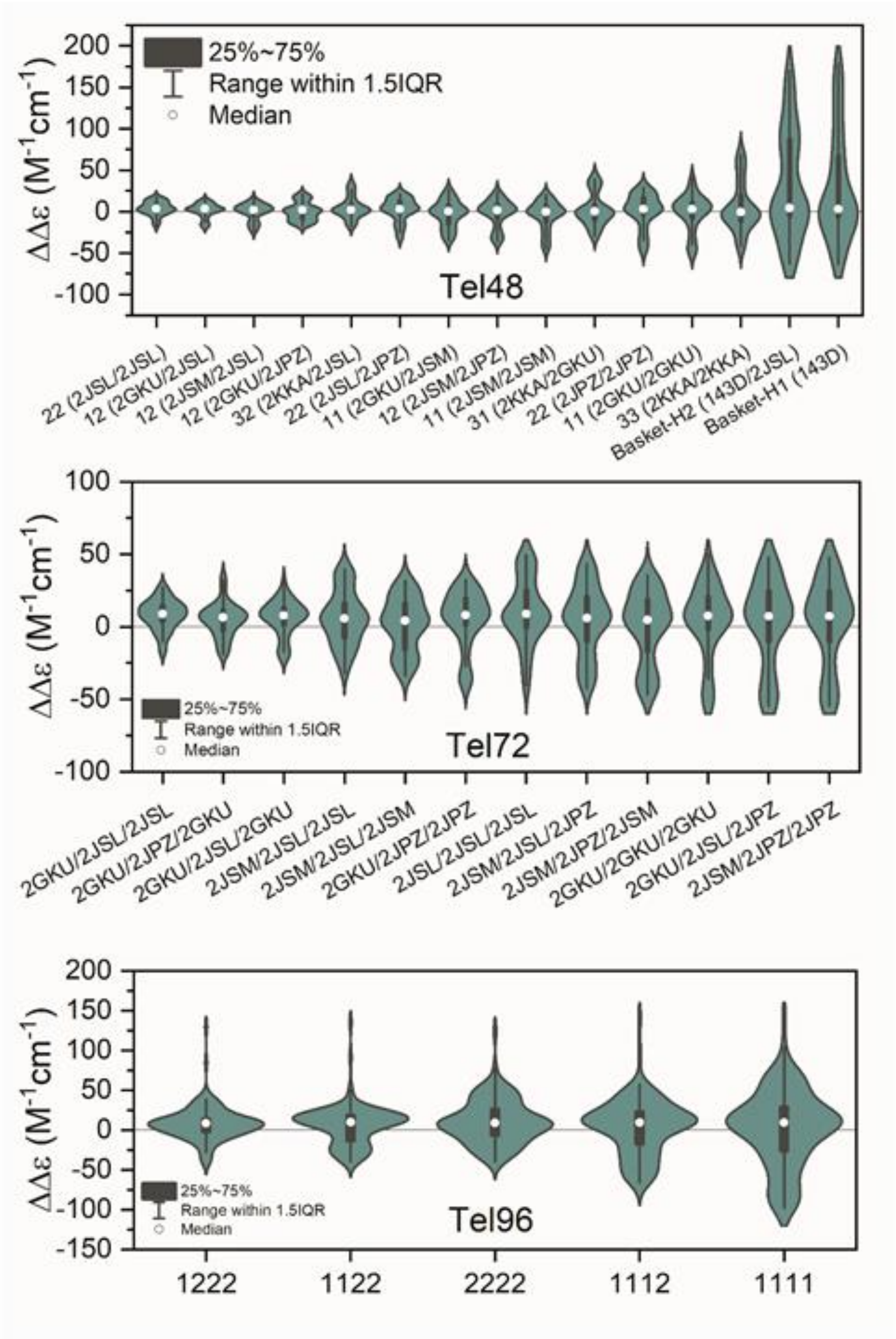

Figure S7. Violin plots of residuals obtained as the difference between experimental and theoretical CD reconstruction curves for Tel48, Tel72, and Tel96. The residuals are plotted from best (left) to worst (right) based on residual sum of squares analysis in Origin 2020.

REFERENCES

1. Petraccone, L., Trent, J.O. and Chaires, J.B. (2008) The tail of the telomere. *J Am Chem Soc*, **130**, 16530-16532.
2. Kirby, N., Cowieson, N., Hawley, A.M., Mudie, S.T., McGillivray, D.J., Kusel, M., Samardzic-Boban, V. and Ryan, T.M. (2016) Improved radiation dose efficiency in solution SAXS using a sheath flow sample environment. *Acta Crystallogr D Struct Biol*, **72**, 1254-1266.
3. Hopkins, J.B., Gillilan, R.E. and Skou, S. (2017) BioXTAS RAW: improvements to a free open-source program for small-angle X-ray scattering data reduction and analysis. *J Appl Crystallogr*, **50**, 1545-1553.
4. Franke, D., Petoukhov, M.V., Konarev, P.V., Panjkovich, A., Tuukkanen, A., Mertens, H.D.T., Kikhney, A.G., Hajizadeh, N.R., Franklin, J.M., Jeffries, C.M. *et al.* (2017) ATSAS 2.8: a comprehensive data analysis suite for small-angle scattering from macromolecular solutions. *J Appl Crystallogr*, **50**, 1212-1225.
